# Squamous Eccrine Ductal Carcinoma Treated With Adjuvant Radiation Therapy

**DOI:** 10.7759/cureus.112769

**Published:** 2026-07-16

**Authors:** Ashley N Austin, Daniel A Jones, William McAfee

**Affiliations:** 1 Radiation Oncology, Philadelphia College of Osteopathic Medicine, Moultrie, USA; 2 Radiation Oncology, Phoebe Putney Memorial Hospital, Albany, USA

**Keywords:** adjuvant radiotherapy, close margins, ductal differentiation, high-risk recurrence, local recurrence, lymphovascular invasion, metastasis, perineural invasion, squamous eccrine ductal carcinoma

## Abstract

Squamous eccrine ductal carcinoma (SEDC) is a rare skin cancer with limited academia or documented definitive and/or adjuvant therapeutic options. Retrospective studies report aggressive course, high risk of local recurrence, and/or distant metastasis. Histopathology describes it to possess both squamous and ductal differentiation, causing diagnostic obscurity and misdiagnosis, begetting underdiagnosis, mottling the prognostic course. If perineural or lymphovascular invasion (LVI) is noted, increased risk of local recurrence is evident. With recurrence risk rates in mind, a physician could infer that wide local excision (WLE) with negative margins or Mohs microscopic surgery (MMS) may not be enough for a cure. After WLE, the role of adjuvant radiotherapy is unclear. In our patient, adjuvant radiotherapy was offered to maximize local control.

## Introduction

Squamous eccrine ductal carcinoma (SEDC) is a rare, infiltrative, cutaneous tumor, with 127 cases reported and published in the literature from 1991 to 2025 [1]. Morphologic features show it to be an intermediate between squamous cell carcinoma (SCC) and sweat gland carcinomas [2], although it is believed to be derived from eccrine glands [1]. SEDC is classified as a sweat gland carcinoma; however, there is debate on whether it should be classified as a variant of SCC [2]. It has also been found that with other reported cases, 70.4-87% of those patients had a personal history of non-melanomatous skin cancers [1,2]. It is suggested that SEDC should be treated with the same approach as lesions with similar histologic features and behavior, such as SCC [3]. Local recurrence (LR) rates after wide local excision (WLE) and Mohs microscopic surgery (MMS) have been reported to be 18-80% [2-6] and 0-12.5% [3,4,6], respectively. Additionally, a metastasis risk of 13-50% [3-6] and a mortality rate of up to 5% [6] have been reported. Other factors that increase LR are positive margins with perineural invasion (PNI) [4,6] and lymphovascular invasion (LVI) [6].

## Case presentation

A 75-year-old man presented with a rapidly growing lesion on the upper midline chest over the course of two months. It was reported to have been approximately 1.5 cm grossly. Subsequently, he underwent WLE (Figures 1-2). Pathology was consistent with SEDC. There was no evidence of PNI or LVI. Deep margins were negative by 2 mm. His postoperative course was uneventful. Staging studies failed to demonstrate regional or distant disease. The biopsy histopathology can be appreciated in Figures 3-5.

**Figure 1:**
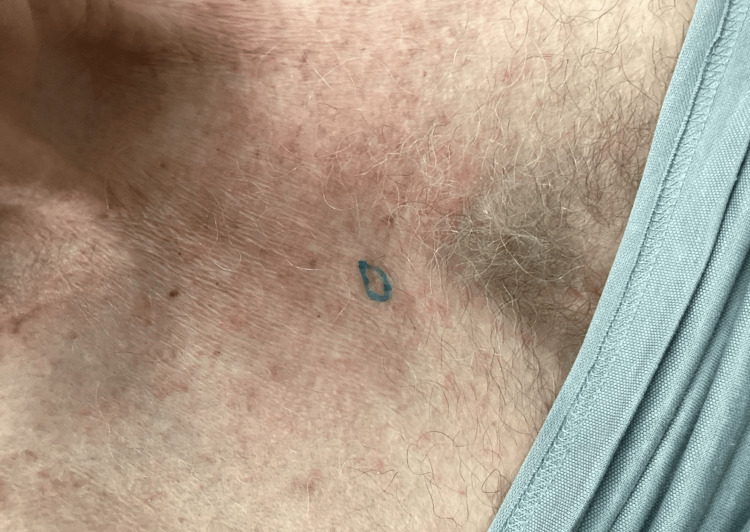
Lesion of the right medial inferior chest before wide local excision (WLE). A marker was used to clearly indicate the area of intended excision.

**Figure 2:**
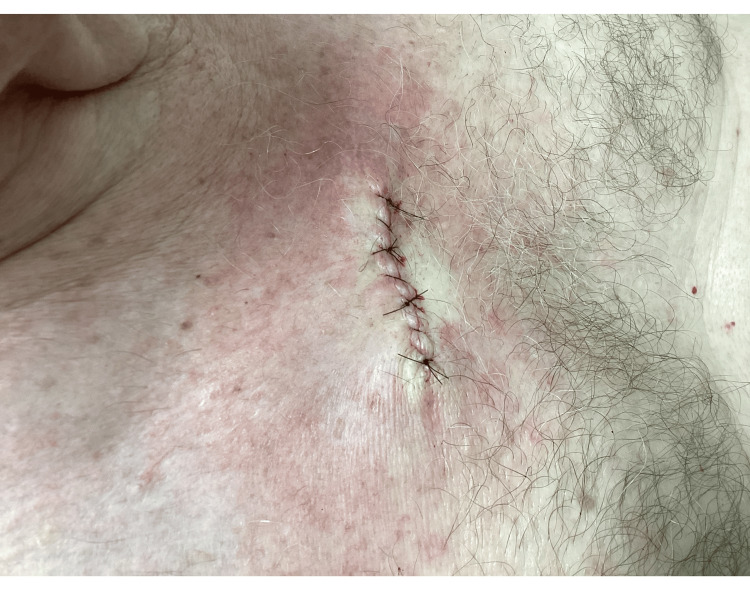
Right medial inferior chest status post wide local excision (WLE) with stitches.

**Figure 3:**
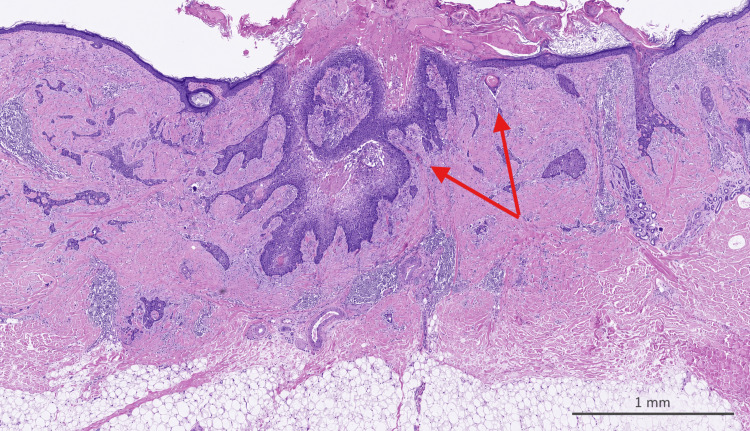
Histopathology of the right medial inferior chest biopsy specimen. Low power shows aggressive infiltration of small tumor islands into the deep dermis (red arrows).

**Figure 4:**
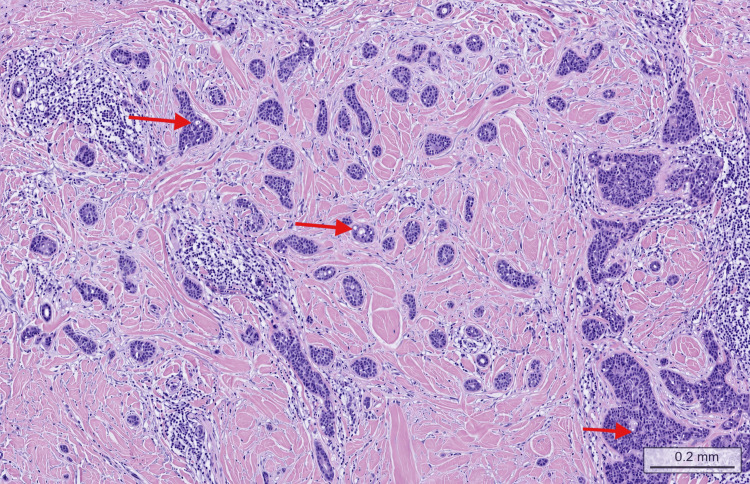
Histopathology of the right medial inferior chest biopsy specimen. High power shows tumor islands with arrows depicting duct formation.

**Figure 5:**
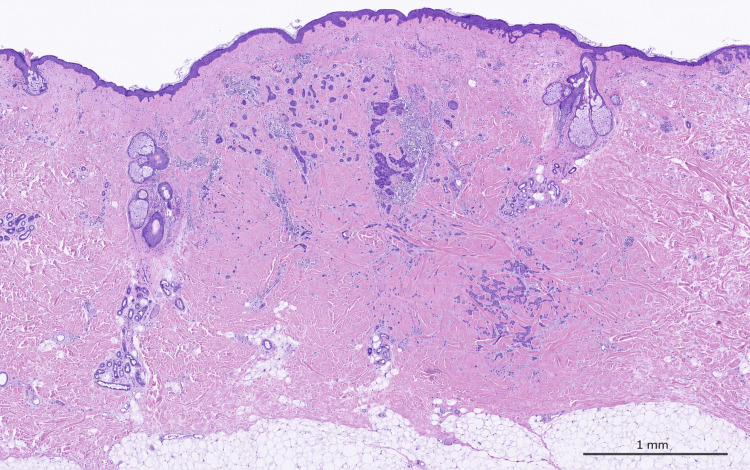
Histopathology of right medial inferior chest biopsy specimen. Low power with biopsy site shows prominent superficial squamatization of the tumor.

Due to the aggressive tumor pathology, close margin, and concern of LR, he was offered adjuvant radiotherapy to improve local control. He received 44 Gy over 10 fractions, delivered with 6 MeV energy with a 1 cm bolus, 2.5 cm margins to the block edge. Dose was verified with a diode. Treatment was tolerated well with a low-grade skin reaction.

He was seen in the follow-up at three months with a normal exam and subsequently at six months with a restaging CT imaging and is disease-free. 

## Discussion

SEDC is a rare, malignant cutaneous cancer; its histopathology will display intermediate morphology of both SCC and sweat gland carcinomas [1,2]. Atypical squamous cells are prominent superficially [3]. There is a deeper component of ductal differentiation, characterized by small lumina, associated with tumor cell atypia that may appear squamoid or cuboidal [2]. Tumor growth typically infiltrates deeply into the dermis and subcutaneous tissue [3], with a desmoplastic stroma [2]. SEDC is currently classified as an adnexal or sweat gland carcinoma; however, it has been debated whether this tumor might be better considered an infiltrative variant of SCC with glandular differentiation [2]. Due to the overlapping histology, it can be difficult to distinguish between SEDC and SCC, making diagnosis difficult [1,2,4,5], posing the additional issue of misdiagnosis as SCC [1,3,4]. Accurate diagnosis of SEDC from SCC is clinically significant because of the lack of risk stratification and fixed clinical management paradigm [2], and increased risk for local recurrence and metastasis [1-6]. Dermatopathologists have found two specific immunohistological stainings (IHS) for eccrine/ductal differentiation [1,2,6], which aid in diagnosis [4] by staining the glandular tissue [3]: carcinoembryonic antigen (CEA) and epithelial membrane antigen (EMA) [1-3,5,6].

SEDC has no established staging system [4], nor does it have finite guidelines for treatment [1,4]. In some literature, it raises the question of treating SEDC the same as other skin cancers with similar histopathology, such as SCC [3]. Per the National Comprehensive Cancer Network (NCCN) risk stratification for SCC, one is considered very high risk (as opposed to low risk or high risk) if one of the following criteria were met: tumor size is >4 cm with pathologic criteria of being poorly differentiated, desmoplastic subtype, invasion depth of >6 mm or invasion beyond subcutaneous fat, present LVI, and PNI of nerves deeper than the dermis or larger than 0.1 mm [7]. Adjuvant radiotherapy was prescribed to this patient with SEDC one month postoperatively from WLE due to the margin status with respect to its known aggressive, infiltrative pathology.

## Conclusions

Due to diagnostic infrequency, pathological diagnosis of SEDC remains somewhat challenging. Additionally, there are no evidence-based guidelines in staging, risk stratification, or treatment for SEDC. It is recommended in medical literature that SEDC should be treated as SCC. We presented this rare case of cutaneous SEDC. After excision, our patient did well with adjunctive radiotherapy and is disease-free on follow-up.
